# Interaction between rheumatoid arthritis and mediterranean diet on the risk of cardiovascular disease for the middle aged and elderly from National Health and Nutrition Examination Survey (NHANES)

**DOI:** 10.1186/s12889-023-15478-1

**Published:** 2023-03-31

**Authors:** Yuwei Zhan, Zhou Yang, Ying Liu, Feng Zhan, Shudian Lin

**Affiliations:** Rheumatology Department, Hainan General Hospital, Hainan Affiliated Hospital of Hainan Medical University, No.19 Xiuhua Road, Xiuying District, Haikou, Hainan Province 570311 P.R. China

**Keywords:** Rheumatoid arthritis, Mediterranean diet, Cardiovascular diseases, Antagonistic effect, NHANES

## Abstract

**Background:**

Cardiovascular diseases (CVD) occurrence were associated with rheumatoid arthritis (RA) and Mediterranean diet (MD), but few studies have been conducted to explore the combined effect. This study was to outline the relationship of coexistence of RA and MD on the risk of CVD based on the National Health and Nutrition Examination Survey (NHANES) database.

**Methods:**

The data of this cross-sectional study was from the NHANES 2005–2010. The definition of CVD and RA was based on the self-reported questions, respectively; and the alternate MD Index assessed all participants’ adherence to the MD. Weighted multivariate logistic regression was adopted to explore the relationship of RA, MD on the risk of CVD, and coexistence effect of RA and MD. The additive interaction was evaluated by the relative excess risk due to interaction (RERI), attributable proportion (AP) and the synergy index (SI). The multiplicative interaction was evaluated by odds ratio (OR) and 95% confidence interval (CI) of product-term.

**Results:**

A total of 3,352 participants from NHANES database who were divided into CVD group (n = 385) and non-CVD group (n = 2,967). The result indicated that RA (Model 1: OR = 3.98, 95%CI: 2.76–5.73; Model 2: OR = 2.65, 95%CI: 1.69–4.16) and low adherence to the MD (Model 1: OR = 1.82, 95%CI: 1.13–2.93; Model 2: OR = 1.67, 95%CI:1.01–2.77) was associated with an increased risk of CVD, respectively. Additionally, we observed the additive (RERI = 4.76, 95% CI: 0.52-9.00; AP = 0.74, 95% CI: 0.54–0.95; SI = 8.21, 95% CI: 1.48–45.51) and multiplicative (OR = 3.63, 95% CI: 1.44–9.15) interaction of RA and low adherence to the MD on the risk of CVD.

**Conclusion:**

RA and MD were associated with CVD occurrence, respectively, and there may be an interaction between RA and MD for the development of CVD.

**Supplementary Information:**

The online version contains supplementary material available at 10.1186/s12889-023-15478-1.

## Background

Nowadays, cardiovascular diseases (CVD) are still considered to be the leading cause of death and disability globally [1]. According to the Global Burden of Disease (GBD) study, the global prevalence of CVD was estimated to have increased by 93% over the past three decades, up to 523 million in 2019 [2], which placed a significant economic burden on health systems and communities. Therefore, exploring the prevention and risk factors of CVD is very important for the management of CVD in future.

It is well known that inflammation plays an important role in the development of CVD [3–5]. Rheumatoid arthritis (RA) is a chronic autoimmune inflammatory disease with a global prevalence ratio of about 0.5-1%, which caused damage both joints and extra-articular organs [6, 7]. A number of studies have reported that RA was associated with an increased CVD risk [8, 9]. Løgstrup, et al., demonstrated that patients with RA were more likely to suffer from the risk of heart failure than the general population [10]. Not only that, the risk of CVD was also influenced by lifestyle factors, such as smoking, physical activity and poor diet. Studies suggested that diet may increase the risk of CVD by regulating metabolic pathways and homeostasis [11, 12]. Over the past few years, Mediterranean diet (MD), which has been characterized by high intake of fish, olive oil, fruits, vegetables, whole grains, legumes/nuts, and moderate alcohol consumption, was considered to have an anti-inflammatory effect and one of the best cardiovascular health diets [11].

Recently, a study also found that MD could reduce pain and increase physical function of patients with RA [13], implicating that there might be a combined effect of RA and MD on the risk of CVD. However, to date, there were few studies focusing on the interaction analysis between RA and MD. Thus, our study aims to outline the relationship of coexistence of RA and MD on the risk of CVD for the middle aged and elderly. We believe that the research would be useful reference for reducing the risk of CVD in patients with RA.

## Methods

### Study population

The study population came from the National Health and Nutritional Examination Survey (NHANES) 2005-2010. NHANES database is a program of studies performed by the Centers for Disease Control and Prevention (CDC) of America using a multistage, probability sampling methods [14], which examined a nationally representative sample of approximately 5,000 people from 15 different counties each year ( https://www.cdc.gov/nchs/nhanes/about_nhanes.htm).

**Fig. 1:**
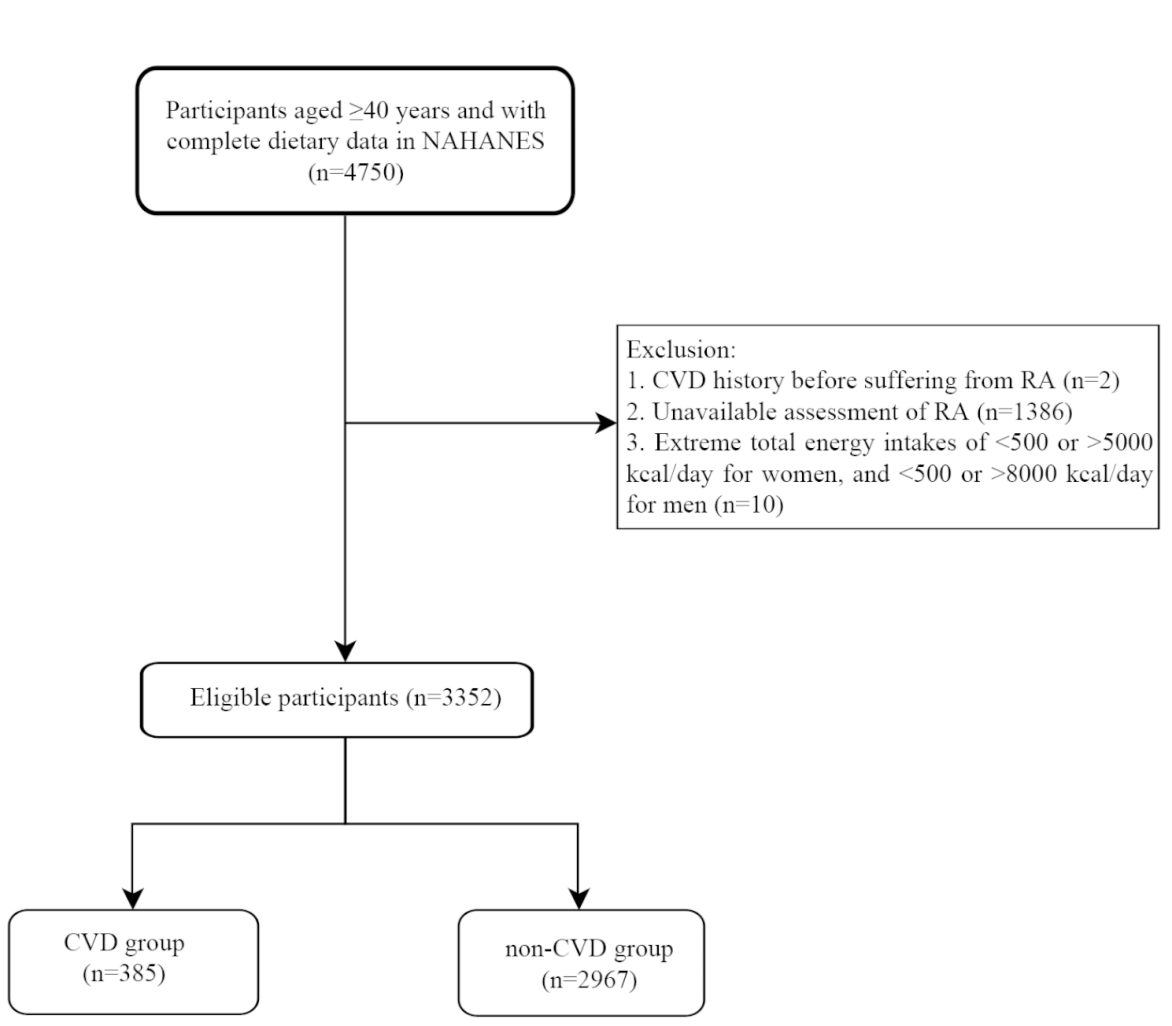
Participant flowchart of the study

This cross-sectional study extracted 4,750 middle aged and elderly participants (aged ≥ 40 years old) [15] and with complete dietary data from the NHANES database 2005–2010. Exclusion criteria were as following: (1) participants had CVD before suffering from RA (n = 2); (2) participants had unavailable assessment of RA (n = 1,386); (3) participants had extreme total energy intakes of < 500 or > 5000 kcal/day for women, and < 500 or > 8000 kcal/day for men (n = 10). Finally, a total of 3,352 participants were included in this study, and they were divided into CVD group (n = 385) and non-CVD group (n = 2,967) (Fig. 1). The requirement of ethical approval for this was waived by the Institutional Review Board of Hainan Affiliated Hospital of Hainan Medical University, because the data was accessed from NHANES (a publicly available database). All methods were carried out in accordance with relevant guidelines and regulations (declaration of Helsinki). All individuals provided written informed consent before participating in the study.

### Data collection

This study collected some variables [9, 13]: age (years), gender, ethnicity, educational level, marital status, poverty-income ratio (PIR), waist circumference (cm), body mass index (BMI, kg/m^2^), smoking, drinking, physical activity [metabolic equivalent (MET)·min/week], hypertension, diabetes, dyslipidemia, family history of CVD, RA, MD score, energy (kcal/day/kg), protein (%), carbohydrate (%), total-sugar (%), total-fat (%), C-reactive protein (CRP, mg/dL), uric acid (mg/dL), estimated glomerular filtration rate (eGFR, mL/min/1.73m^2^).

Waist circumference was classified as abdominal non-obese (< 88 cm for women and < 102 cm for men) and abdominal obese (≥ 88 cm for women and ≥ 102 cm for males) [16]. MET is used to describe the energy consumption when performing a specific activity [17], and physical activity was calculated as weekly energy consumption in this study, weekly energy consumption (MET·min/week) = MET × exercise time of corresponding activity (min)/week. Energy (kcal/day/kg) is defined as total energy (kcal/day) divided by the weight (kg). Hypertension was defined as systolic blood pressure ≥ 140 mmHg or diastolic blood pressure ≥ 90 mmHg or self-reported hypertension. Diabetes was defined as fasting glucose ≥ 7.0mmol/L or self-reported diabetes. Dyslipidemia was determined by total cholesterol ≥ 200 mg/dL or triglyceride ≥ 150 mg/dL or low-density lipoprotein cholesterol ≥ 130 mg/dL or high-density lipoprotein cholesterol ≤ 40 mg/dL. The data of uric acid was collected by timed endpoint method used the Beckman Synchron LX20 or Beckman Coulter UniCel® DxC800, both monitored for absorbance changes at 520 nm. eGFR was calculated by the following equation: 141×min [serum creatinine (Scr)/κ, 1] ^α^×max (Scr/κ, 1)^− 1.029^ × 0.993^age^×1.108 (if female) ×1.159 (if black), κ is 0.7 for females and 0.9 for males, α is − 0.329 for females and − 0.411 for males, min indicates the minimum of Scr/κ or 1, and max indicates the maximum of Scr/κ or 1 [18].

### Measurements

#### Assessment of Mediterranean diet

We adopted the alternate Mediterranean Diet Index (aMD) to assess all participants’ adherence to the MD. The aMD scores was determined by assessing the intakes of alcohol, presumed detrimental foods [including red and processed meat], and presumed beneficial foods [seafood, whole grains, legumes, nuts, fruits, vegetables (except potatoes), and the ratio of monounsaturated fish, and the ratio of monounsaturated fatty acids-to-saturated fatty acids] according to the information on 24 h dietary recall questionnaires, which with a total of 9 points [19]. A score of 1 point was assigned to participants who had an intake of presumed beneficial foods higher than the median level, and presumed detrimental foods lower the median level. For alcohol, “1 point” was assigned to participants who had moderate alcohol consumption (10–25 g/day for men and 5–15 g/day for women); “0 points” was assigned for participants who had not a consumption of meeting the aforementioned criteria. The higher the aMD scores, the better of the adherence to the MD. We divided the MD score into two groups based on the median [20]: <3.80 as low adherence to the MD group, and ≥ 3.80 as high adherence to the MD group in this study.

#### Assessment of rheumatoid arthritis

The diagnosis of RA was based on the self-reported information: “Has a doctor or other health professional ever told you that you had RA?”. The individuals would be diagnosed as having RA if the question were answered as “yes” [21].

#### Outcomes

The outcome of this study was considered as the incident of CVD. The definition of CVD was based on the self-reported questions: Ever told you had congestive heart failure/ coronary heart disease/angina/angina pectoris/heart attack/stroke. Participants who answered “yes” to the question were defined as having CVD [22].

### Statistical analysis

Considered the complex sampling design of the NHANES database, we used a weighted analysis: the sampling weights for interview (WTMEC2YR) and study design variables (SDMVPSU and SDMVSTRA). The mean ± standard error (SE) was used to describe the distributed variables, and comparison between the CVD group and non-CVD group was performed by Student’s t-test. The number of cases and the composition ratio [n (%)] was used to describe the categorical data, and comparison between two groups adopted Rao-Scott Chi-square test. SPSS (version 23.0) and R (version 4.1.2) software was used for statistical analyses.

In the present study, we conducted a weighted univariate logistic regression to screen for confounding factors. Then, weighted multivariate logistic regression was adopted to explore the relationship of RA, MD on the risk of CVD, respectively. Two models were used in this study. Model 1: adjusted age, gender and ethnicity; Model 2: adjusted covariates that were statistically significant in the univariate logistic regression analysis, including age, gender, ethnicity, educational level, PIR, waist circumference, smoking, physical activity, hypertension, diabetes, dyslipidemia, family history of CVD, uric acid, eGFR and energy (Supplementary Table 1). Odds ratio (OR) and 95% confidence interval (CI) were calculated. In addition, we assessed the coexistence effect of RA and MD for the risk of CVD by weighted multivariate regression analysis. The additive interaction between RA and MD on the risk of CVD was evaluated by the relative excess risk due to interaction (RERI), attributable proportion (AP) and the synergy index (SI) [23]. When the 95% CI of RERI or AP were not included 0, or the 95% CI of SI not contained 1, we considered there was an additive interaction. The multiplicative interaction between RA and MD on the risk of CVD was evaluated by OR, when the 95% CI of product-term was not contained 1, we considered there was a multiplicative interaction. Additionally, we also performed subgroup analyses based on age, gender, ethnicity, BMI and family history of CVD, to explore the interaction between RA and MD on the risk of CVD in different population. Statistical significance difference was confirmed by *P* < 0.05. For the samples with missing data, we adopted.

multiple interpolation methods, and sensitivity analysis was performed on the data before and after interpolation (Supplemental Table 2).

## Results

### General characteristics of participants

Table 1 displays the general characteristics of participants in CVD group and non-CVD group, and there were some variables with a significant difference between the CVD group and non-CVD group in the distribution of age, gender, educational level, PIR, waist circumference, BMI, smoking, physical activity, hypertension, diabetes, family history of CVD, CRP, uric acid, eGFR, energy. Participants in the CVD group had a lower PIR and MD score, and a higher waist circumference and BMI compared to those who were non-CVD group. Additionally, RA might be more common in the CVD group than non-CVD group. The detailed information was shown in Table 1.

**Table 1:** General characteristics of included participants

Variables	Total (n = 3352)	Non-CVD group (n = 2967)	CVD group (n = 385)	Statistics	*P*
Age, years, Mean (S.E.)	53.97 (0.48)	52.88 (0.48)	65.55 (0.86)	t=-13.26	< 0.001
Gender, n (%)				χ^2^ = 28.141	< 0.001
Female	1796 (51.92)	1531 (50.24)	265 (69.75)		
Male	1556 (48.08)	1436 (49.76)	120 (30.25)		
Ethnicity, n (%)				χ^2^ = 1.407	0.495
Non-Hispanic White	1761 (77.15)	1527 (77.12)	234 (77.40)		
Non-Hispanic Black	649 (9.13)	570 (9.00)	79 (10.55)		
Other Race	942 (13.72)	870 (13.88)	72 (12.05)		
Educational level, n (%)				χ^2^ = 6.396	0.011
High School and below	1611 (36.38)	1406 (35.60)	205 (44.66)		
University or above	1741 (63.62)	1561 (64.40)	180 (55.34)		
Marital status [n (%)]				χ^2^ = 5.622	0.060
Married	2070 (68.59)	1853 (69.10)	217 (63.20)		
Never married	232 (5.14)	211 (5.31)	21 (3.35)		
Others*	1050 (26.27)	903 (25.59)	147 (33.45)		
PIR, Mean (S.E.)	3.52 (0.08)	3.58 (0.08)	2.96 (0.13)	t = 4.92	< 0.001
Waist circumference, cm, Mean (S.E.)	98.33 (0.45)	97.50 (0.50)	107.07 (0.93)	t=-8.46	< 0.001
Waist circumference, n (%)				χ^2^ = 39.202	< 0.001
Abdominal non-obese	1462 (46.89)	1361 (49.00)	101 (24.46)		
Abdominal obese	1890 (53.11)	1606 (51.00)	284 (75.54)		
BMI, kg/m^2^, Mean (S.E.)	28.31 (0.17)	28.14 (0.19)	30.10 (0.39)	t=-4.13	< 0.001
BMI, n (%)				χ^2^ = 22.160	< 0.001
<25 kg/m^2^	984 (32.10)	904 (33.25)	80 (19.89)		
25 kg/m^2^ ~ 30 kg/m^2^	1238 (37.30)	1102 (37.62)	136 (33.92)		
≥30 kg/m^2^	1130 (30.60)	961 (29.13)	169 (46.19)		
Smoking, n (%)				χ^2^ = 40.147	< 0.001
No	1720 (53.37)	1581 (55.19)	139 (33.99)		
Yes	1632 (46.63)	1386 (44.81)	246 (66.01)		
Drinking, n (%)				χ^2^ = 0.272	0.602
No	941 (23.60)	840 (23.40)	101 (25.81)		
Yes	2411 (76.40)	2127 (76.60)	284 (74.19)		
Physical activity, n (%)				χ^2^ = 23.925	< 0.001
<450 MET· min/week	785 (28.62)	712 (29.16)	73 (22.86)		
≥450 MET· min/week	1714 (52.11)	1546 (52.72)	168 (45.66)		
Unknown	853 (19.27)	709 (18.13)	144 (31.48)		
Hypertension, n (%)				χ^2^ = 44.124	< 0.001
No	1771 (61.45)	1683 (64.55)	88 (28.41)		
Yes	1581 (38.55)	1284 (35.45)	297 (71.59)		
Diabetes, n (%)				χ^2^ = 131.867	< 0.001
No	2835 (89.95)	2576 (91.56)	259 (72.90)		
Yes	517 (10.05)	391 (8.44)	126 (27.10)		
Dyslipidemia, n (%)				χ^2^ = 3.377	0.066
No	2263 (70.28)	2038 (71.11)	225 (61.53)		
Yes	1089 (29.72)	929 (28.89)	160 (38.47)		
Family history of CVD, n (%)				χ^2^ = 6.105	0.013
No	2876 (85.15)	2586 (86.00)	290 (76.10)		
Yes	476 (14.85)	381 (14.00)	95 (23.90)		
CRP, mg/dL, Mean (S.E.)	0.36 (0.03)	0.34 (0.03)	0.53 (0.06)	t=-3.09	0.003
Uric acid, mg/dL, Mean (S.E.)	5.51 (0.04)	5.44 (0.04)	6.25 (0.13)	t=-6.30	< 0.001
eGFR, mL/min/1.73m^2^, Mean (S.E.)	96.97 (0.66)	98.27 (0.70)	83.18 (1.42)	t = 9.28	< 0.001
Energy, n (%)				χ^2^ = 14.440	< 0.001
< 25.33 kcal/day/kg	1843 (49.98)	1576 (48.27)	267 (68.10)		
≥25.33 kcal/day/kg	1509 (50.02)	1391 (51.73)	118 (31.90)		
Protein, %, Mean (S.E)	16.63 (0.20)	16.67 (0.22)	16.17 (0.44)	t = 0.91	0.366
Carbohydrate, %, Mean (S.E)	47.68 (0.34)	47.59 (0.35)	48.67 (0.94)	t=-1.13	0.263
Total sugars, %, Mean (S.E)	20.92 (0.32)	20.83 (0.33)	21.90 (0.63)	t=-1.58	0.121
Total fat, %, Mean (S.E)	34.20 (0.33)	34.18 (0.34)	34.41 (0.68)	t=-0.34	0.737
RA, n (%)				χ^2^ = 92.893	< 0.001
No	3017 (93.01)	2735 (94.61)	282 (75.93)		
Yes	335 (6.99)	232 (5.39)	103 (24.07)		
MD, Mean (S.E)	4.53 (0.11)	4.58 (0.12)	4.06 (0.26)	t = 1.80	0.078
MD, n (%)				χ^2^ = 3.353	0.067
High adherence to the MD group	2191 (64.47)	1976 (65.32)	215 (55.48)		
Low adherence to the MD group	1161 (35.53)	991 (34.68)	170 (44.52)		

CVD, cardiovascular diseases; GED, General Educational Development; AA, Associate of Arts; PIR, poverty-income ratio; BMI, body mass index; CRP, C-reactive protein; eGFR, estimated glomerular filtration rate; RA, rheumatoid arthritis; MD, Mediterranean Diet; S. E., Standard error

Others*: widowed, divorced, separated and living with partner

### The association of RA, MD and the risk of CVD

The association of RA and CVD, MD and CVD were presented in the Table 2. The result of multivariate logistic regression model indicated that RA was associated with an increased risk of CVD (Model 1: OR = 3.98, 95%CI: 2.76–5.73, *P* < 0.001; Model 2: OR = 2.65, 95%CI: 1.69–4.16, *P* < 0.001). Compared to those who were high adherence to the MD, those who were low adherence to the MD were positively associated with the risk of CVD [OR of 1.82 (95% CI: 1.13–2.93, *P* = 0.015)] in Model 1 and OR of 1.67 (95% CI: 1.01–2.77, *P* = 0.046) in Model 2.

**Table 2:** The association of RA and CVD, MD and CVD by weighted multivariate logistic regression

Variables	Model 1		Model 2	
	OR (95% CI)	*P*	OR (95% CI)	*P*
RA				
No	Ref		Ref	
Yes	3.98 (2.76–5.73)	< 0.001	2.65 (1.69–4.16)	< 0.001
MD				
High adherence to the MD group	Ref		Ref	
Low adherence to the MD group	1.82 (1.13–2.93)	0.015	1.67 (1.01–2.77)	0.046

CVD, cardiovascular diseases; RA, rheumatoid arthritis; MD, Mediterranean Diet; Ref, reference; OR, odds ratio; CI, confidence interval.

Model 1: adjusted age, gender and ethnicity;

Model 2: adjusted age, gender, ethnicity, educational level, poverty-income ratio, waist circumference, smoking, physical activity, hypertension, diabetes, dyslipidemia, family history of cardiovascular diseases, uric acid, estimated glomerular filtration rate and energy.

### The coexistence of RA and MD on the risk of CVD

As shown in Table 3, we established the interaction term of RA and MD to assess the additive interaction: RA & low adherence to the MD, non-RA & low adherence to the MD, RA & high adherence to the MD, non-RA & high adherence to the MD. After adjusting confounding factors, we observed an additive interaction of RA and low adherence to the MD on the risk of CVD (RERI = 4.76, 95% CI: 0.52-9.00; AP = 0.74, 95% CI: 0.54–0.95; SI = 8.21, 95% CI: 1.48–45.51), which indicated that there might be a synergistic effect.

**Table 3:** The additive interaction of RA and MD on the risk of CVD

RA	MD	OR (95%CI)	*P*	
Yes	Low	6.42 (3.06–13.43)	< 0.001	
No	Low	1.29 (0.76–2.20)	0.345	
Yes	High	1.37 (0.74–2.56)	0.313	
No	High	Ref		
RERI		4.76 (0.52-9.00)		
AP		0.74 (0.54–0.95)		
SI		8.21 (1.48–45.51)		

CVD, cardiovascular diseases; RA, rheumatoid arthritis; MD, Mediterranean Diet; OR: odds ratio; CI: confidence interval; RERI, relative excess risk due to interaction; AP, attributable proportion; SI, synergy index; Ref, reference

Adjusted age, gender, ethnicity, educational level, poverty-income ratio, waist circumference, smoking, physical activity, hypertension, diabetes, dyslipidemia, family history of cardiovascular diseases, uric acid, estimated glomerular filtration rate and energy

Simultaneously, we also assessed a multiplicative interaction of RA and MD on the risk of CVD (Table 4). After incorporating RA, low adherence to the MD and the product-term of RA & low adherence to the MD into multivariate logistic regression model, we found that the multiplicative interaction between RA and low adherence to the MD on the risk of CVD was statistically significant (OR = 3.63, 95% CI: 1.44–9.15), which also indicated that there might be a synergistic effect. Additionally, the statistic of likelihood ratio test was 8.141 (*P* = 0.009), suggesting there was an interaction between RA and MD on CVD risk.

**Table 4:** The multiplicative interaction of RA and low adherence to the MD on the risk of CVD

Variables	OR (95% CI)	*P*
RA	1.37 (0.74–2.56)	0.313
Low adherence to the MD	1.29 (0.76–2.20)	0.345
RA* low adherence to the MD	3.63 (1.44–9.15)	0.007

CVD, cardiovascular diseases; RA, rheumatoid arthritis; MD, Mediterranean Diet; OR: odds ratio; CI: confidence interval;

Adjusted age, gender, ethnicity, educational level, poverty-income ratio, waist circumference, smoking, physical activity, hypertension, diabetes, dyslipidemia, family history of cardiovascular diseases, uric acid, estimated glomerular filtration rate and energy

### The coexistence of RA and MD on the risk of CVD based on age, gender, ethnicity, BMI and family history of CVD

The performed subgroup analyses based on age, gender, ethnicity, BMI and family history of CVD were conducted. As shown in Table 5, the additive interaction between RA and low adherence to the MD on CVD risk was present in the other subgroup analyses except in those with a family history of CVD. Furthermore, Table 6 also indicated the multiplicative interaction of RA and MD on the risk of CVD based on age, gender, ethnicity, BMI and family history of CVD. Significant multiplicative interaction between depression and low adherence to the MD were found among participants who were non-Hispanic white, aged ≥ 60 years, had not a family history of CVD, and had a BMI of 25–30 kg/m^2^.

**Table 5:** The additive interaction of RA and MD on the risk of CVD for different populations

RA	MD		OR (95%CI)	*P*	OR (95%CI)	*P*	OR (95%CI)	*P*
Subgroup I: Age			Age < 60 years		Age ≥ 60 years			
No	High		Ref		Ref			
No	Low		1.68 (0.63–4.46)	0.290	1.15 (0.65–2.04)	0.633		
Yes	High		1.18 (0.24–5.83)	0.839	1.44 (0.68–3.03)	0.331		
Yes	Low		6.57 (1.53–28.09)	0.012	6.71 (2.73–16.49)	< 0.001		
RERI			4.71 (-3.59-13.01)		5.12 (-0.30-10.54)			
AP			0.72 (0.27–1.16)		0.76 (0.54–0.98)			
SI			6.50 (0.29-143.87)		9.74 (1.14–83.05)			
Subgroup II: Gender			Male		Female			
No	High		Ref		Ref			
No	Low		1.32 (0.65–2.69)	0.439	1.67 (0.83–3.36)	0.149		
Yes	High		2.00 (0.71–5.57)	0.183	1.34 (0.59–3.04)	0.481		
Yes	Low		5.61 (1.73–18.14)	0.005	6.72 (3.33–13.58)	< 0.001		
RERI			3.30 (-2.88-9.48)		4.72 (0.23–9.21)			
AP			0.59 (0.05–1.13)		0.70 (0.41–0.99)			
SI			3.51 (0.49–25.28)		5.70 (0.97–33.51)			
Subgroup III: Ethnicity			Non-Hispanic White		Non-Hispanic Black		Others	
No		High	Ref		Ref		Ref	
No		Low	1.02 (0.56–1.86)	0.937	2.74 (1.55–4.87)	0.001	4.45 (2.00-9.89)	< 0.001
Yes		High	1.00 (0.41–2.41)	0.999	5.15 (1.58–16.84)	0.008	1.21 (0.32–4.58)	0.778
Yes		Low	5.54 (2.23–13.81)	< 0.001	6.29 (2.06–19.24)	0.002	16.34 (2.95–90.34)	0.002
RERI			4.521 (0.03–9.01)		-0.60 (-8.11-6.91)		11.68 (-15.04-38.41)	
AP			0.815 (0.61–1.02)		-0.10 (-1.35-1.16)		0.72 (0.22–1.21)	
SI			194.025 (0.00-6.706E24)		0.90 (0.23–3.47)		4.20 (0.62–28.49)	
Subgroup IV: BMI			<25 kg/m^2^		25 kg/m^2^ ~ 30 kg/m^2^		≥ 30 kg/m^2^	
No		High	Ref		Ref		Ref	
No		Low	3.43 (1.51–7.78)	0.004	1.09 (0.59–2.01)	0.768	1.15 (0.55–2.42)	0.698
Yes		High	1.52 (0.27–8.41)	0.626	1.14 (0.47–2.77)	0.773	1.73 (0.76–3.95)	0.189
Yes		Low	9.19 (2.67–31.59)	< 0.001	7.58 (1.62–35.44)	0.011	6.27 (1.99–19.80)	0.002
RERI			5.24 (-6.55-17.04)		6.35 (-4.77-17.47)		4.39 (-2.10-10.88)	
AP			0.57 (-0.11-1.25)		0.84 (0.57–1.10)		0.70 (0.35–1.05)	
SI			2.78 (0.41–18.77)		28.40 (0.09-8600.86)		5.97 (0.81–44.26)	
Subgroup V: Family of CVD			No		Yes			
No	High		Ref		Ref			
No	Low		1.07 (0.61–1.90)	0.805	3.18 (1.27–7.98)	0.015		
Yes	High		2.43 (1.16–5.09)	0.020	0.27 (0.06–1.16)	0.077		
Yes	Low		7.39 (3.13–17.45)	< 0.001	5.49 (0.95–31.79)	0.057		
RERI			4.89 (-0.78-10.56)		3.04 (-6.17-12.24)			
AP			0.66 (0.36–0.96)		0.55 (-0.28-1.39)			
SI			4.26 (1.17–15.50)		3.09 (0.23–40.91)			

CVD, cardiovascular diseases; RA, rheumatoid arthritis; MD, Mediterranean Diet; OR: odds ratio; CI: confidence interval; BMI, body mass index; RERI, relative excess risk due to interaction; AP, attributable proportion; SI, synergy index; Ref, reference

Subgroup I: Adjusted gender, ethnicity, educational level, poverty-income ratio (PIR), waist circumference, smoking, physical activity, hypertension, diabetes, dyslipidemia, family history of CVD, uric acid, eGFR and energy

Subgroup II: Adjusted age, ethnicity, educational level, PIR, waist circumference, smoking, physical activity, hypertension, diabetes, dyslipidemia, family history of CVD, uric acid, eGFR and energy

Subgroup III: Adjusted age, gender, educational level, PIR, waist circumference, smoking, physical activity, hypertension, diabetes, dyslipidemia, family history of CVD, uric acid, eGFR and energy

Subgroup IV: Adjusted age, gender, ethnicity, educational level, PIR, waist circumference, smoking, physical activity, hypertension, diabetes, dyslipidemia, family history of CVD, uric acid, eGFR and energy

Subgroup V: Adjusted age, gender, ethnicity, educational level, PIR, waist circumference, smoking, physical activity, hypertension, diabetes, dyslipidemia, uric acid, eGFR and energy

**Table 6:** The multiplicative interaction of RA and MD on the risk of CVD for different populations

Variables	OR (95%CI)	*P*	OR (95%CI)	*P*	OR (95%CI)	*P*
Subgroup I: Age	Age < 60 years		Age ≥ 60 years			
RA	1.18 (0.24–5.83)	0.839	1.44 (0.68–3.03)	0.331		
Low adherence to the MD	1.68 (0.63–4.46)	0.290	1.15 (0.65–2.04)	0.633		
RA* low adherence to the MD	3.32 (0.42–26.39)	0.250	4.06 (1.42–11.62)	0.010		
Subgroup II: Gender	Male		Female			
RA	2.00 (0.71–5.57)	0.183	1.34 (0.59–3.04)	0.481		
Low adherence to the MD	1.32 (0.65–2.69)	0.439	1.67 (0.83–3.36)	0.149		
RA* low adherence to the MD	2.13 (0.49–9.34)	0.307	3.02 (0.86–10.55)	0.082		
Subgroup III: Ethnicity	Non-Hispanic White		Non-Hispanic Black		Others	
RA	1.00 (0.41–2.41)	0.999	5.15 (1.58–16.84)	0.008	1.21 (0.32–4.58)	0.778
Low adherence to the MD	1.02 (0.56–1.86)	0.937	2.74 (1.55–4.87)	0.001	4.45 (2.00-9.89)	< 0.001
RA* low adherence to the MD	5.42 (1.69–17.37)	0.005	0.45 (0.09–2.17)	0.306	3.05 (0.36–25.84)	0.299
Subgroup IV: BMI	<25 kg/m^2^		25 kg/m^2^ ~ 30 kg/m^2^		≥ 30 kg/m^2^	
RA	1.52 (0.27–8.41)	0.626	1.14 (0.47–2.77)	0.773	1.73 (0.76–3.95)	0.189
Low adherence to the MD	3.43 (1.51–7.78)	0.004	1.09 (0.59–2.01)	0.768	1.15 (0.55–2.42)	0.698
RA* low adherence to the MD	1.76 (0.16–19.28)	0.635	6.09 (1.09–33.89)	0.040	3.14 (0.86–11.45)	0.081
Subgroup V: Family of CVD	No		Yes			
RA	2.43 (1.16–5.09)	0.020	0.27 (0.06–1.16)	0.077		
Low adherence to the MD	1.07 (0.61–1.90)	0.805	3.18 (1.27–7.98)	0.015		
RA* low adherence to the MD	2.84 (1.07–7.50)	0.036	6.32 (0.60–66.90)	0.122		

CVD, cardiovascular diseases; RA, rheumatoid arthritis; MD, Mediterranean Diet; OR: odds ratio; CI: confidence interval; BMI, body mass index;

Subgroup I: Adjusted gender, ethnicity, educational level, poverty-income ratio (PIR), waist circumference, smoking, physical activity, hypertension, diabetes, dyslipidemia, family history of CVD, uric acid, eGFR and energy

Subgroup II: Adjusted age, ethnicity, educational level, PIR, waist circumference, smoking, physical activity, hypertension, diabetes, dyslipidemia, family history of CVD, uric acid, eGFR and energy

Subgroup III: Adjusted age, gender, educational level, PIR, waist circumference, smoking, physical activity, hypertension, diabetes, dyslipidemia, family history of CVD, uric acid, eGFR and energy

Subgroup IV: Adjusted age, gender, ethnicity, educational level, PIR, waist circumference, smoking, physical activity, hypertension, diabetes, dyslipidemia, family history of CVD, uric acid, eGFR and energy

Subgroup V: Adjusted age, gender, ethnicity, educational level, PIR, waist circumference, smoking, physical activity, hypertension, diabetes, dyslipidemia, uric acid, eGFR and energy

## Discussion

In this study, we verified that RA was associated with an increased risk of CVD, and low adherence to the MD was related to the risk of CVD. An important finding of our study was that the coexistence of RA and low adherence to the MD might be related to the risk of CVD, and there was a synergistic effect.

Previous studies have reported that RA is an inflammatory joint disease, and patients with RA are at higher risk of atherosclerotic CVD, stroke, heart failure, and atrial fibrillation compared to the general population [24, 25]. A review also concluded that treatment of RA may reduce the risk of CVD by reducing chronic inflammation [25]; in other words, the risk of CVD is strongly associated with chronic inflammation in RA disease. Recently, MD has been suggested to be beneficial in preventing and improving CVD [26]. In the study of Critselis E, et al., they found that high adherence to the MD was associated with a lower 10-year risk of fatal and non-fatal CVD [27]. Consistent with established studies [25, 27], our study also indicated the risk of CVD for individuals with independent RA was 2.65 times than non-RA individuals, and the risk of CVD decreased as adherence to the MD increased. As pointed out by Johansson et al., [28] there was an inverse association between MD and RA risk among only men or in seropositive RA. The underlying mechanisms might be that MD’s bioactive compounds decreased the blood pressure, lipids and inflammatory markers.

But, the joint effects of RA and MD on the occurrence of CVD were rarely studied. In the current study, the result indicated that participants with both RA & low adherence to the MD was associated a higher risk of CVD. There was a synergistic effect between RA and low adherence to the MD for the development of CVD. In addition, we found that both additive and multiplicative interactions of RA and low adherence to MD on CVD risk in the analysis of different subgroups were distinguished. The multiplicative interaction was only found among participants who were non-Hispanic white, aged ≥ 60 years, had not a family history of CVD, and had a BMI of 25–30 kg/m^2^. It is worth noting that neither the additive nor multiplicative interactions of RA and low adherence MD on CVD risk were statistically different in the subgroup with a family history of CVD, which may be related to the small sample size (n = 476).

There were also underlying mechanisms to support the interaction between RA and adherence to the MD on the occurrence of CVD. Mechanisms linking of both RA and the MD on the occurrence of CVD may include shared pathway. Firstly, inflammatory mediators might be a pathway linking of the RA and MD to the risk of CVD [29]. Systemic inflammation is considered as a significant factor in the increased CVD risk among patients with RA [30]. MD patterns rich is in fruits, vegetables, polyunsaturated fatty acids and other nutrients, presenting an anti-inflammatory property, which might feature a protective role for the development of CVD among patients with RA [31]. Secondly, RA is characterized by endothelial dysfunction which tends to be the indicator for CVD occurrence [29]. But the polyphenols in the MD patterns could have a positive effect on endothelial dysfunction [32]. Additionally, oxidative stress is a hallmark of systemic inflammation in RA, and could promote the insulin resistance and further exacerbate the imbalance of reactive oxygen species (ROS) and antioxidants [33], which contributed to the development of CVD. However, MD patterns are associated with a lower level of oxidative stress [34]. When patients with RA have a high adherence to the MD, MD patterns could decrease the level of oxidative stress among patients with RA, which in turn further reduce the risk of CVD occurrence.

This is the first cross-sectional study based on the NHANES database to investigate the interaction between RA and adherence to the MD for the development of CVD. We believe that knowing the interaction of RA & the adherence to the MD might be useful for reducing the risk of CVD in patients with RA. However, some limitations have to be mentioned. Firstly, the assessments of RA, adherence to the MD, and CVD in the NHANES database were based on the self-reported information for participants, which may be subject to recall bias. Nevertheless, several studies have used the self-reported information to examine the factors related to CVD risk [35]. Secondly, we excluded some participants who had unavailable assessment of RA, and we cannot be sure if the excluded participants affect the result. Lastly, this study was a cross-sectional study that could not confirm a causal relationship of RA and adherence to the MD and the risk of CVD. A further study with more focus on the causal relationship is therefore suggested.

## Conclusion

RA and MD were associated with CVD occurrence, respectively, and there may be an interaction between RA and MD for the development of CVD. We believe that knowing the interaction of RA and adherence to the MD might be useful for the risk of CVD in patients with RA. However, more prospective clinical studies are needed to further validate our results and explore the possible mechanisms.

## Electronic supplementary material

Below is the link to the electronic supplementary material.

Supplementary Material 1

## Data Availability

The datasets used and/or analyzed during the current study are available from the NHANES database, https://wwwn.cdc.gov/nchs/nhanes/.

## List of abbreviations

CVD: Cardiovascular diseases
GBD: Global Burden of Disease
RA: Rheumatoid arthritis
MD: Mediterranean diet
NHANES: National Health and Nutritional Examination Survey
CDC: Centers for Disease Control and Prevention
PIR: Poverty-income ratio
BMI: Body mass index
CRP: C-reactive protein
aMD: Alternate Mediterranean Diet Index
OR: Odds ratio
CI: Confidence interval
RERI: Relative excess risk due to interaction
AP: Attributable proportion
SI: Synergy index

## References

[1] Masaebi F, Salehi M, Kazemi M, Vahabi N, Azizmohammad Looha M, Zayeri F (2021). Trend analysis of disability adjusted life years due to cardiovascular diseases: results from the global burden of disease study 2019. BMC Public Health.

[2] Roth GA, Mensah GA, Johnson CO, Addolorato G, Ammirati E, Baddour LM (2020). Global Burden of Cardiovascular Diseases and Risk factors, 1990–2019: Update from the GBD 2019 study. J Am Coll Cardiol.

[3] Schunk SJ, Kleber ME, März W, Pang S, Zewinger S, Triem S (2021). Genetically determined NLRP3 inflammasome activation associates with systemic inflammation and cardiovascular mortality. Eur Heart J.

[4] Shivappa N, Godos J, Hébert JR, Wirth MD, Piuri G, Speciani AF et al. Dietary Inflammatory Index and Cardiovascular Risk and Mortality-A Meta-Analysis.Nutrients. 2018;10. 10.3390/nu10020200 PMC5852776 29439509

[5] Jia C, Anderson JLC, Gruppen EG, Lei Y, Bakker SJL, Dullaart RPF (2021). High-density lipoprotein anti-inflammatory capacity and Incident Cardiovascular events. Circulation.

[6] Ahmed S, Jacob B, Carsons SE, De Leon J, Reiss AB. Treatment of Cardiovascular Disease in Rheumatoid Arthritis: A Complex Challenge with Increased Atherosclerotic Risk.Pharmaceuticals (Basel). 2021;15. 10.3390/ph15010011 PMC8778152 35056068

[7] Kerola AM, Rollefstad S, Semb AG (2021). Atherosclerotic Cardiovascular Disease in Rheumatoid Arthritis: impact of inflammation and Antirheumatic Treatment. Eur Cardiol.

[8] Kašperová S, Tarabčáková L, Kašperová B, Šteňová EK (2021). Rheumatoid arthritis and metabolic disorders. Vnitr Lek.

[9] Wilton KM, Achenbach SJ, Davis JM, Myasoedova E, Matteson EL, Crowson CS (2021). Erectile Dysfunction and Cardiovascular Risk in Men with Rheumatoid Arthritis: a Population-based Cohort Study. J Rheumatol.

[10] Løgstrup BB, Ellingsen T, Pedersen AB, Darvalics B, Olesen KKW, Bøtker HE (2021). Cardiovascular risk and mortality in rheumatoid arthritis compared with diabetes mellitus and the general population. Rheumatology (Oxford).

[11] Widmer RJ, Flammer AJ, Lerman LO, Lerman A (2015). The Mediterranean diet, its components, and cardiovascular disease. Am J Med.

[12] Rees K, Takeda A, Martin N, Ellis L, Wijesekara D, Vepa A (2019). Mediterranean-style diet for the primary and secondary prevention of cardiovascular disease. Cochrane Database Syst Rev.

[13] Forsyth C, Kouvari M, D’Cunha NM, Georgousopoulou EN, Panagiotakos DB, Mellor DD (2018). The effects of the Mediterranean diet on rheumatoid arthritis prevention and treatment: a systematic review of human prospective studies. Rheumatol Int.

[14] Li S, Sun W, Zhang D (2019). Association of Zinc, Iron, copper, and Selenium Intakes with low cognitive performance in older adults: a cross-sectional study from National Health and Nutrition Examination Survey (NHANES). J Alzheimers Dis.

[15] Liu X, Lin Q, Fan K, Tang M, Zhang W, Yang B, Ou Q (2021). The effects of genetic polymorphisms of APOE on circulating lipid levels in middle-aged and elderly chinese Fujian Han population: toward age- and sex-personalized management. Lipids Health Dis.

[16] Grundy SM, Cleeman JI, Daniels SR, Donato KA, Eckel RH, Franklin BA (2005). Diagnosis and management of the metabolic syndrome: an American Heart Association/National Heart, Lung, and Blood Institute Scientific Statement. Circulation.

[17] Jeong SW, Kim SH, Kang SH, Kim HJ, Yoon CH, Youn TJ, Chae IH (2019). Mortality reduction with physical activity in patients with and without cardiovascular disease. Eur Heart J.

[18] De Nicola L, Donfrancesco C, Minutolo R, Lo Noce C, Palmieri L, De Curtis A (2015). Prevalence and cardiovascular risk profile of chronic kidney disease in Italy: results of the 2008-12 National Health Examination Survey. Nephrol Dial Transplant.

[19] Chang CY, Lee CL, Liu WJ, Wang JS. Association of Adherence to the Mediterranean Diet with All-Cause Mortality in Subjects with Heart Failure. Nutrients. 2022; 14. 10.3390/nu14040842 PMC8875916 35215491

[20] Huang Q, Jin Y, Reed NS, Ma Y, Power MC, Talegawkar SA (2020). Diet quality and hearing loss among middle-older aged adults in the USA: findings from National Health and Nutrition Examination Survey. Public Health Nutr.

[21] Shi Y, Zhang J, Huang Y (2021). Prediction of cardiovascular risk in patients with chronic obstructive pulmonary disease: a study of the National Health and Nutrition Examination Survey database. BMC Cardiovasc Disord.

[22] Ran L, Chen Q, Zhang J, Tu X, Tan X, Zhang Y (2021). The multimorbidity of hypertension and osteoarthritis and relation with sleep quality and hyperlipemia/hyperglycemia in China’s rural population. Sci Rep.

[23] Zhong VW, Ning H, Van Horn L, Carnethon MR, Wilkins JT, Lloyd-Jones DM (2021). Diet Quality and Long-Term Absolute Risks for Incident Cardiovascular Disease and Mortality. Am J Med.

[24] Kang S, Han K, Jung JH, Eun Y, Kim IY, Hwang J, Koh EM, Lee S, Cha HS, Kim H, Lee J (2022). Associations between Cardiovascular Outcomes and Rheumatoid Arthritis: a Nationwide Population-Based Cohort Study. J Clin Med.

[25] DeMizio DJ, Geraldino-Pardilla LB (2020). Autoimmunity and inflammation link to Cardiovascular Disease Risk in Rheumatoid Arthritis. Rheumatol Ther.

[26] AlAufi NS, Chan YM, Waly MI, Chin YS, Mohd Yusof BN, Ahmad N (2022). Application of Mediterranean Diet in Cardiovascular Diseases and type 2 diabetes Mellitus: Motivations and Challenges. Nutrients.

[27] Critselis E, Kontogianni MD, Georgousopoulou E, Chrysohoou C, Tousoulis D, Pitsavos C (2021). Comparison of the Mediterranean diet and the Dietary Approach Stop Hypertension in reducing the risk of 10-year fatal and non-fatal CVD events in healthy adults: the ATTICA Study (2002–2012). Public Health Nutr.

[28] Johansson K, Askling J, Alfredsson L, Di Giuseppe D (2018). Mediterranean diet and risk of rheumatoid arthritis: a population-based case-control study. Arthritis Res Ther.

[29] England BR, Thiele GM, Anderson DR, Mikuls TR (2018). Increased cardiovascular risk in rheumatoid arthritis: mechanisms and implications. BMJ.

[30] Semb AG, Ikdahl E, Wibetoe G, Crowson C, Rollefstad S (2020). Atherosclerotic cardiovascular disease prevention in rheumatoid arthritis. Nat Rev Rheumatol.

[31] Gioia C, Lucchino B, Tarsitano MG, Iannuccelli C, Di Franco M. Dietary Habits and Nutrition in Rheumatoid Arthritis: Can Diet Influence Disease Development and Clinical Manifestations?Nutrients. 2020;12. 10.3390/nu12051456 PMC7284442 32443535

[32] Stromsnes K, Mas-Bargues C, Gambini J, Gimeno-Mallench L. Protective Effects of Polyphenols Present in Mediterranean Diet on Endothelial Dysfunction. Oxid Med Cell Longev. 2020; 2020: 2097096. 10.1155/2020/2097096 PMC7428938 32831990

[33] Quiñonez-Flores CM, González-Chávez SA, Del Río Nájera D, Pacheco-Tena C. Oxidative Stress Relevance in the Pathogenesis of the Rheumatoid Arthritis: A Systematic Review. Biomed Res Int. 2016; 2016: 6097417. 10.1155/2016/6097417 PMC4906181 27340664

[34] Aleksandrova K, Koelman L, Rodrigues CE (2021). Dietary patterns and biomarkers of oxidative stress and inflammation: a systematic review of observational and intervention studies. Redox Biol.

[35] Kim DH, Sabour S, Sagar UN, Adams S, Whellan DJ (2008). Prevalence of hypovitaminosis D in cardiovascular diseases (from the National Health and Nutrition Examination Survey 2001 to 2004). Am J Cardiol.

