# Supplementary material for: Interaction between rheumatoid arthritis and mediterranean diet on the risk of cardiovascular disease for the middle aged and elderly from National Health and Nutrition Examination Survey (NHANES)
Source: BMC Public Health. 2023 Mar 31;23:620. doi: 10.1186/s12889-023-15478-1 (PMC10067192; doi:10.1186/s12889-023-15478-1)
Supplement: Supplementary file 1 — Supplementary Material 1 [file 12889_2023_15478_MOESM1_ESM.docx]

**Supplementary Table 1 The screening of confounding factors by a weighted univariate logistic regression**

| **Variables** | **OR (95%CI)** | ***P*** |
| --- | --- | --- |
| Age | 1.09 (1.08-1.11) | <0.001 |
| Gender |  |  |
| Female | Ref |  |
| Male | 0.44 (0.32-0.60) | <0.001 |
| Ethnicity |  |  |
| Non-Hispanic White | Ref |  |
| Non-Hispanic Black | 1.17 (0.80-1.70) | 0.409 |
| Others | 0.87 (0.58-1.30) | 0.477 |
| Educational level |  |  |
| High School and below | Ref |  |
| University or above | 0.68 (0.50-0.93) | 0.018 |
| Marital status |  |  |
| Married | Ref |  |
| Never married | 0.69 (0.35-1.35) | 0.273 |
| Others* | 1.43 (0.95-2.16) | 0.087 |
| PIR | 0.78 (0.71-0.86) | <0.001 |
| Waist circumference |  |  |
| Abdominal non-obese | Ref |  |
| Abdominal obese | 2.97 (2.07-4.26) | <0.001 |
| Smoking |  |  |
| No | Ref |  |
| Yes | 2.39 (1.77-3.23) | <0.001 |
| Drinking |  |  |
| No | Ref |  |
| Yes | 0.88 (0.53-1.45) | 0.604 |
| Physical activity |  |  |
| <450 MET· min/week | Ref |  |
| ≥450 MET· min/week | 1.10 (0.76-1.60) | 0.593 |
| Unknown | 2.21 (1.38-3.54) | 0.001 |
| Hypertension |  |  |
| No | Ref |  |
| Yes | 4.59 (2.76-7.64) | <0.001 |
| Diabetes |  |  |
| No | Ref |  |
| Yes | 4.03 (3.01-5.40) | <0.001 |
| Dyslipidemia |  |  |
| No | Ref |  |
| Yes | 1.54 (0.96-2.48) | 0.075 |
| Family history of CVD |  |  |
| No | Ref |  |
| Yes | 1.93 (1.12-3.31) | 0.018 |
| CRP | 1.16 (0.97-1.40) | 0.109 |
| Uric acid | 1.51 (1.34-1.70) | <0.001 |
| eGFR | 0.96 (0.95-0.97) | <0.001 |
| Energy |  |  |
| < 25.33 kcal/day/kg | Ref |  |
| ≥25.33 kcal/day/kg | 0.44 (0.28-0.69) | <0.001 |
| Protein | 0.22 (0.01-6.95) | 0.381 |
| Carbohydrate | 3.50 (0.39-31.81) | 0.259 |
| Total sugars | 4.44 (0.69-28.51) | 0.114 |
| Total fat | 1.48 (0.14-15.39) | 0.737 |

CVD, cardiovascular diseases; GED, General Educational Development; AA, Associate of Arts; PIR, poverty-income ratio; BMI, body mass index; CRP, C-reactive protein; RA, rheumatoid arthritis; MD, Mediterranean Diet; Others*: widowed, divorced, separated and living with partner; OR, odds ratio; CI, confidence interval.

**Supplemental Table 2 Sensitivity analysis of missing data before and after interpolation**

| **Variables** | **Before the interpolation** | **After the interpolation** | **Statistics** | ***P*** |
| --- | --- | --- | --- | --- |
| Education, n (%) |  |  | χ^2^=1.84 | 0.175 |
| High School and below | 1609 (36.37) | 1611 (36.38) |  |  |
| University or above | 1741 (63.63) | 1741 (63.62) |  |  |
| Waist circumference, cm, Mean (S.E) | 98.32 (0.45) | 98.33 (0.45) | t=-0.24 | 0.815 |
| Smoking, n (%) |  |  | χ^2^=0.97 | 0.325 |
| No | 1720 (53.37) | 1720 (53.37) |  |  |
| Yes | 1631 (46.63) | 1632 (46.63) |  |  |
| Drinking, n (%) |  |  | χ^2^=0.26 | 0.611 |
| No | 898 (23.50) | 941 (23.60) |  |  |
| Yes | 2327 (76.50) | 2411 (76.40) |  |  |
